# Supplementary material for: PAWH1 and PAWH2 are plant-specific components of an Arabidopsis endoplasmic reticulum-associated degradation complex
Source: Nat Commun. 2019 Aug 2;10:3492. doi: 10.1038/s41467-019-11480-7 (PMC6677890; doi:10.1038/s41467-019-11480-7)
Supplement: Supplementary file 1 — Supplementary Information [file 41467_2019_11480_MOESM1_ESM.pdf]

PAWH1 and PAWH2 are plant-specific components of an Arabidopsis endoplasmic reticulum-associated degradation complex

Lin et al.

Supplementary Information

This file contains Supplementary Figure 1-25 with figure legends, Supplementary Table 1 (the primer list) and Supplementary References.

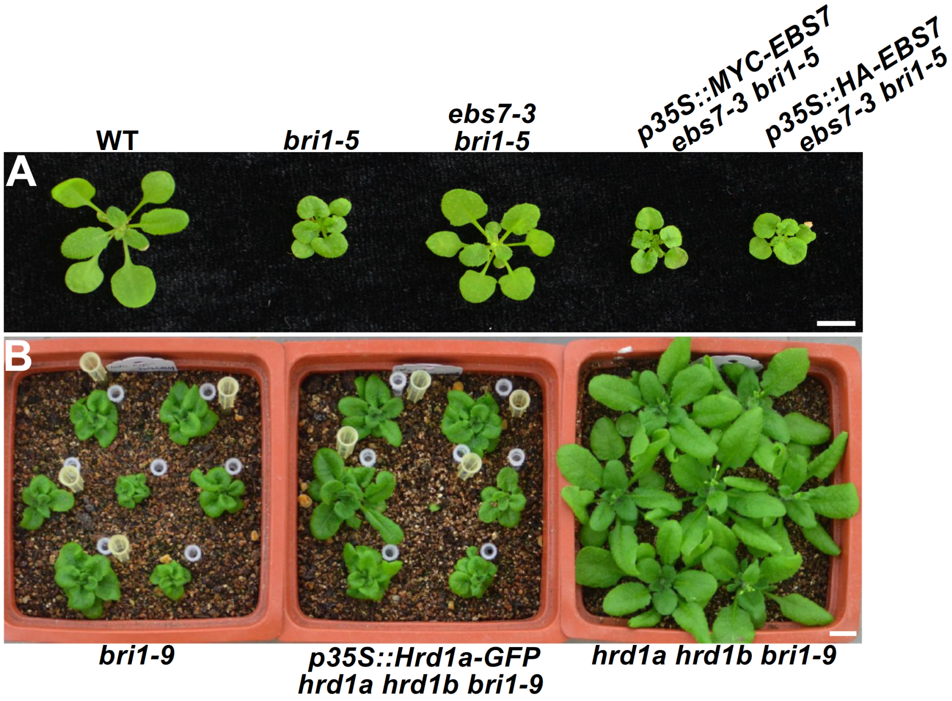

Supplementary Fig. 1. The MYC/HA-tagged EBS7 and GFP-tagged Hrd1a are physiologically active. **a.** Photographs of 3-week-old light-grown seedlings. **b**. Photographs of 5-week-old soil-grown plants. Scale bar = 1 cm.

a

Hrd1a-GFP (IPed from total proteins)

protein	score	coverage	unique peptides
Hrd1a	618	42.1	16
PAWH1	479	74.0	21
PAWH2	467	64.9	23
EBS7	276	30.9	6
EBS5	137	17.1	10
Hrd1b	134	13.0	3
EBS6	50	8.9	2

b

Hrd1a-GFP (IPed from microsomes)

protein	score	coverage	unique peptides
Hrd1a	549	27.6	10
EBS7	130	36.4	4
EBS5	72	3.5	2
PAWH2	69	19.5	5
PAWH1	58	16.5	4

c

HA-EBS7 (IPed from total proteins)

protein	score	coverage	unique peptides
EBS7	855	55.7	8
Hrd1a	500	26.2	8
PAWH1	266	18.6	5
Hrd1b	256	18.5	7
PAWH2	173	17.7	5
EBS5	135	8.3	4
EBS6	108	6.7	2

d

MYC-EBS7 (IPed from total proteins)

protein	score	coverage	unique peptides
EBS7	855	45.4	9
Hrd1a	590	25.2	9
Hrd1b	352	19.1	6
PAWH1	257	18.6	5
PAWH2	173	16.3	4
EBS5	137	9.1	5
EBS6	137	6.7	2

Supplementary Fig. 2. Identification of ERAD components by 4 independent immunoprecipitation-mass spectrometry (IP-MS) experiments. **a-d**. Shown here are 4 tables that list the protein names, mascot scores, amino acid coverages, and the numbers of unique peptides of a given protein identified by LC-MS/MS. It is important to note that the three IP-MS experiments (**a**, **c**, and **d**) with total proteins also identified Hrd1b and EBS6 that were not recovered in the anti-GFP immunoprecipitate with the microsomal preparation (**b**). The source data of the LC-MS/MS experiments can be accessed at https://www.ebi.ac.uk/pride/archive with the dataset identifier PXD013400.

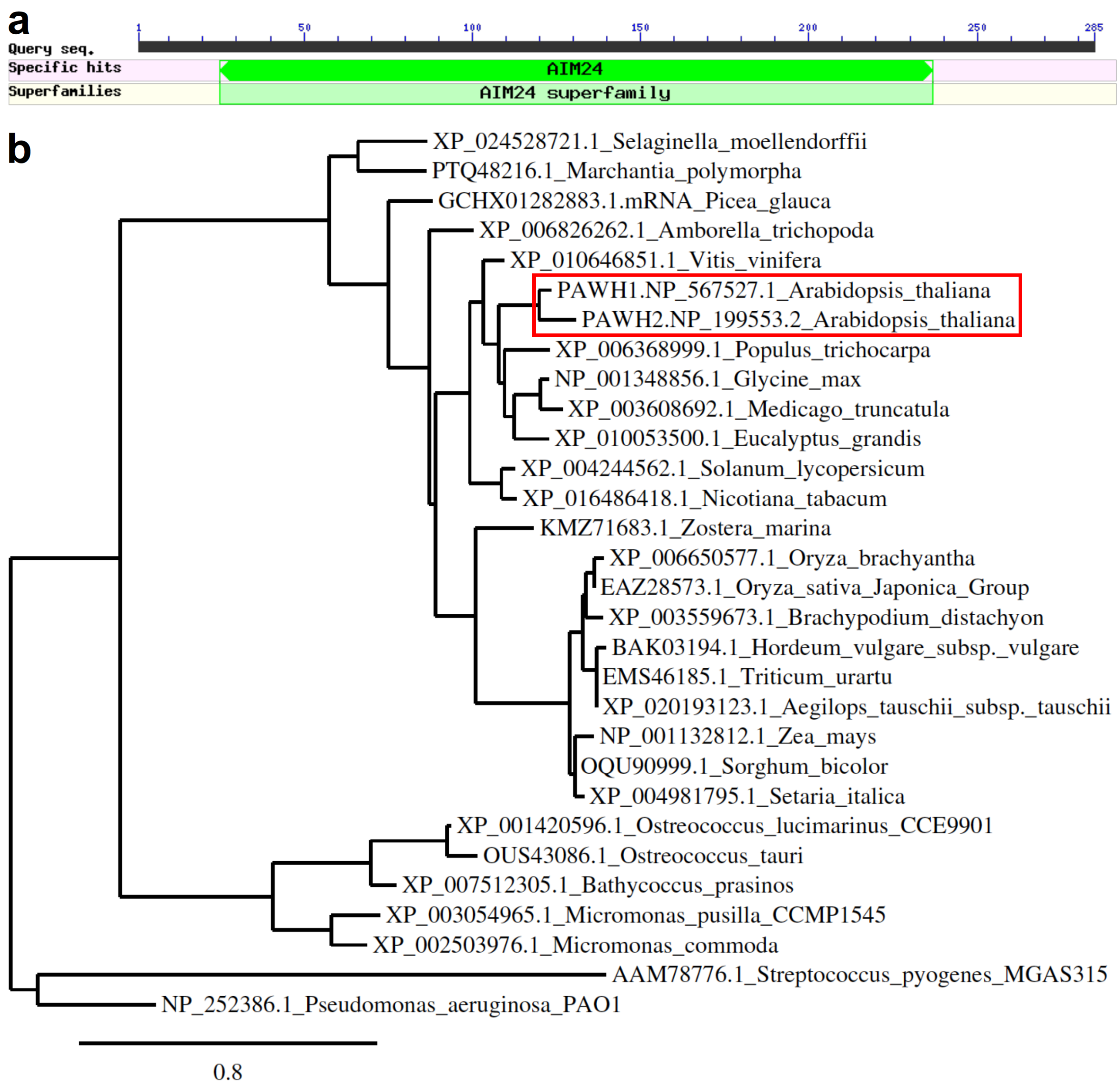

Supplementary Fig. 3. Sequence analysis of PAWH1/2 and their homologs. **a.** A summary diagram of the BLAST search using PAWH1 as the enquiry, revealing AIM24 as a major domain hit, which is a common structural domain shared by the so-called AIM24 superfamily. **b**. Phylogeny analysis of the two PAWH proteins with representative PAWH1/2 homologs from green plants and green algae and the two bacterial proteins, PA3696 of *Pseudomonas aeruginosa* (accession number: AAM78776) and SpyM3_0169 of *Streptococcus pyogenes* (accession number: NP_252386). Shown in the phylogeny tree are the protein accession numbers and the corresponding species names with the exception of the PAWH1/2 homolog of *Picea glauca*, which was translated from an mRNA (accession number: GCHX01282883) identified by a transcriptome study[1]. The protein sequences were obtained from GenBank and subsequently used for the phylogeny analysis at www.phylogeny.fr[2] via the "One Click mode".

a

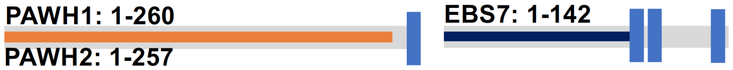

b

AD-fusion	BD-fusion
AD-PAWH1/2	BD-EBS7-N142
AD-PAWH1/2	BD
AD	BD-EBS7-N142
AD-EBS7-N142	BD-PAWH1/2
AD-EBS7-N142	BD
AD	BD-PAWH1/2

c

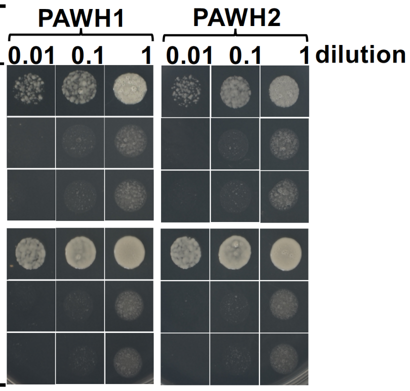

Supplementary Fig. 4. The yeast two hybrid assays of the EBS7-PAWH1/2 interaction. **a**. The top shows the diagram of the fragments of PAWH1/2 (left side, indicated by orange line) and EBS7 (right, indicated by the dark-blue line) used for the yeast two hybrid assay. The vertical blue bars indicate predicted transmembrane domains. **b**. The table shows the co-expressed fusion proteins of PAWH1/2 and EBS7 with the DNA-binding domain (BD) or activation domain (AD) of GAL4 in yeast cells. **c**. Photographs of serially-diluted yeast cells co-expressing various combinations (shown in **b**) of AD/BD fusion proteins on synthetic medium that lacks leucine, tryptophan, and histidine.

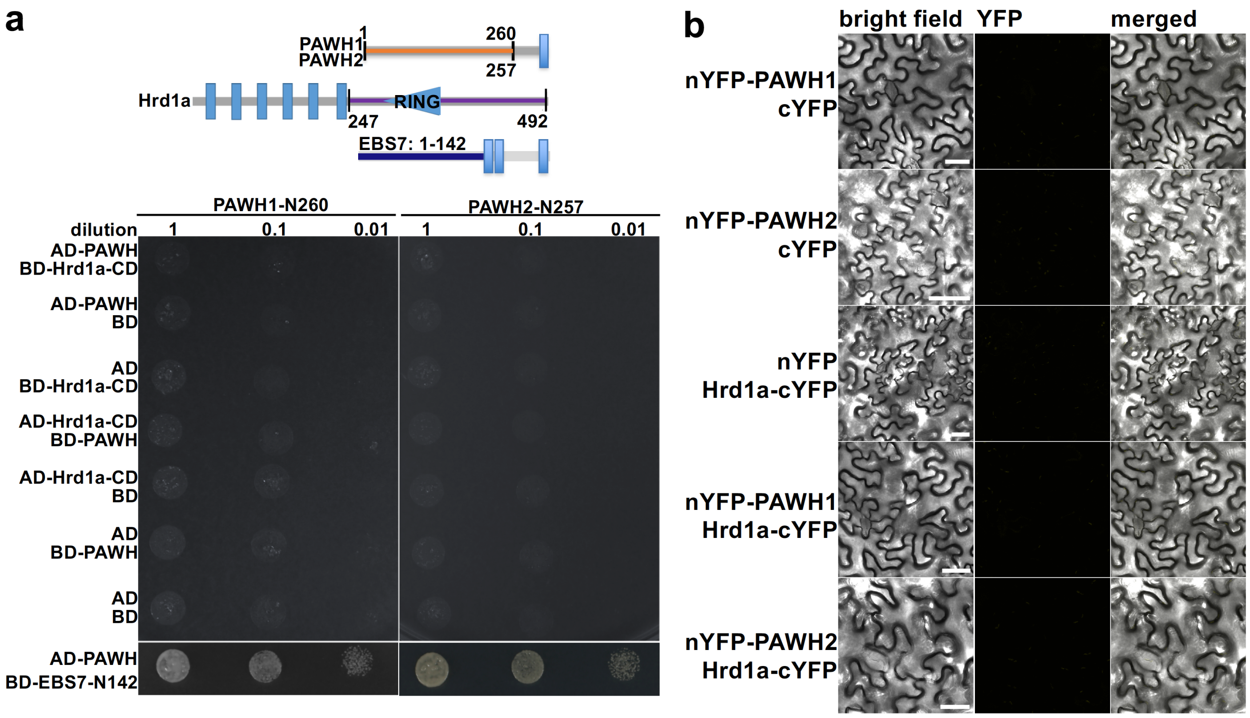

Supplementary Fig. 5. No direct interaction between PAWH1/2 and Hrd1a. **a**. The yeast two-hybrid assays of the PAWH1/2-Hrd1a interaction. The top shows the diagrams of the PAWH1/2, Hrd1a, and EBS7 fragments used for the assays. The bottom shows the images of yeast cells coexpressing various combinations of fusion proteins of Gal4's DNA-binding domain (BD) or activation domain (AD) with the N-terminal fragments of PAWH1/PAWH2 (PAWH1-N260/PAWH2-N257) or with the cytosolic domain of Arabidopsis Hrd1a (Hrd1a-CD). The 10-fold serially-diluted yeast cells were spotted on dropout synthetic medium that lacks leucine, tryptophan, and histidine. Serially-diluted yeast cells coexpressing fusion proteins of AD-PAWH1-N260/PAWH2-N257 and BD-EBS7-N142 were used as the positive control. **b**. Bright field, fluorescent, and superimposed confocal images of tobacco leaf epidermal cells coexpressing the N-terminal (nYFP) and C-terminal (cYFP) yellow fluorescent protein (YFP) fragments fused with or without the full-length PAWH1/2 or Hrd1a. The label on the left indicates the fusion proteins coexpressed in examined tobacco leaf cells. Scale bar = 50 μm.

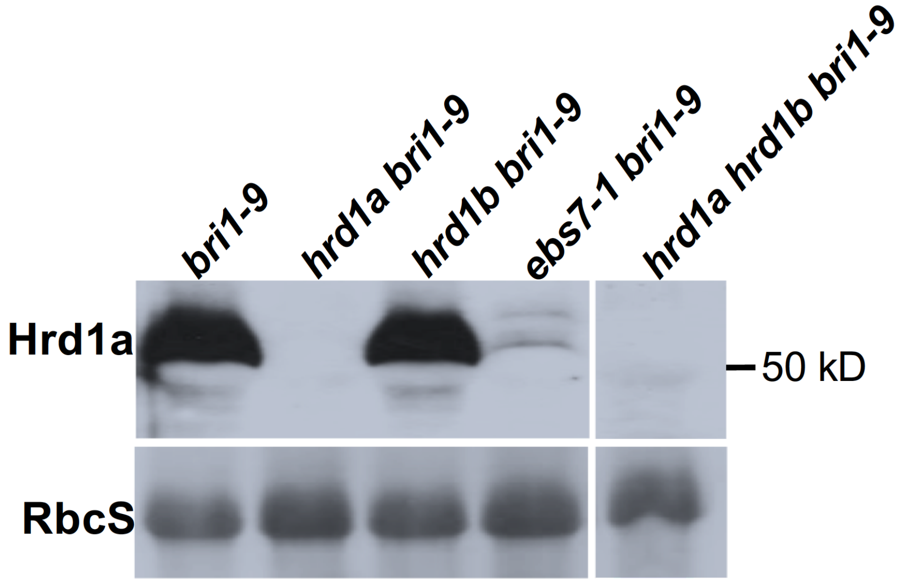

Supplementary Fig. 6. The specificity test of the anti-Hrd1a antibody. Total proteins extracted from 10-day-old light-grown seedlings were separated by 12% SDS-PAGE and analyzed by immunoblotting with the custom-made anti-Hrd1a antibody. The lower strip shows the Ponceau Red-stained RbcS bands used as the loading control. It is important to note that the sample of the last lane was run on the same gel and that the white vertical lines indicate digital removal of two lanes from the blot images. Source data are provided as a Source Data file.

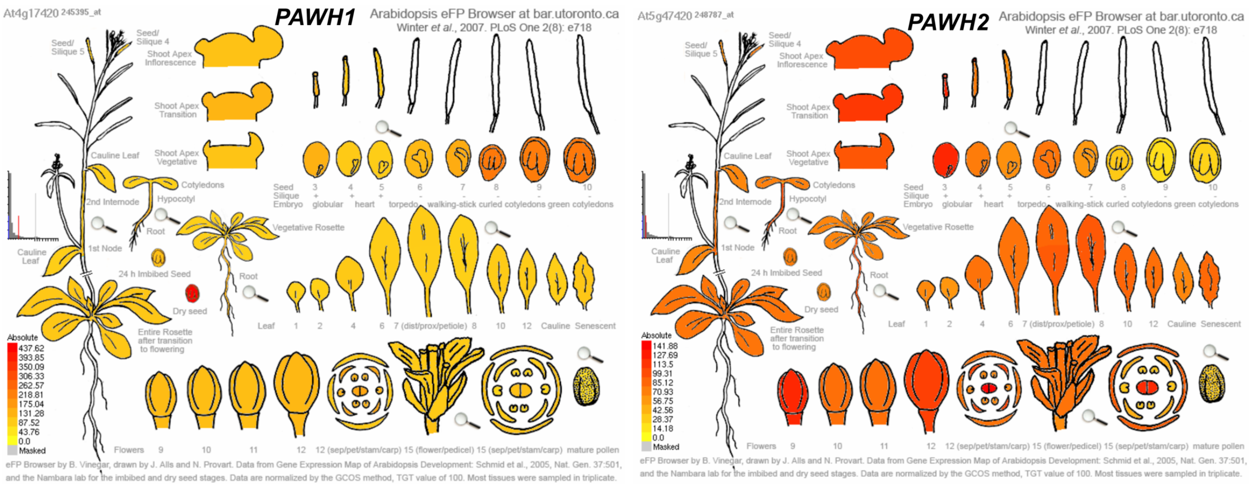

Supplementary Fig. 7. Both *PAWH1* and *PAWH2* are widely expressed in various plant tissues. The two diagrams indicate the gene expression profiles of *PAWH1* (*At4g17420* on the left) and *PAWH2* (*At5g47420* on the right) in different tissues of growing Arabidopsis plants, which were obtained from the Arabidopsis eFP browser web site http://bar.utoronto.ca/efp/cgi-bin/efpWeb.cgi[3].

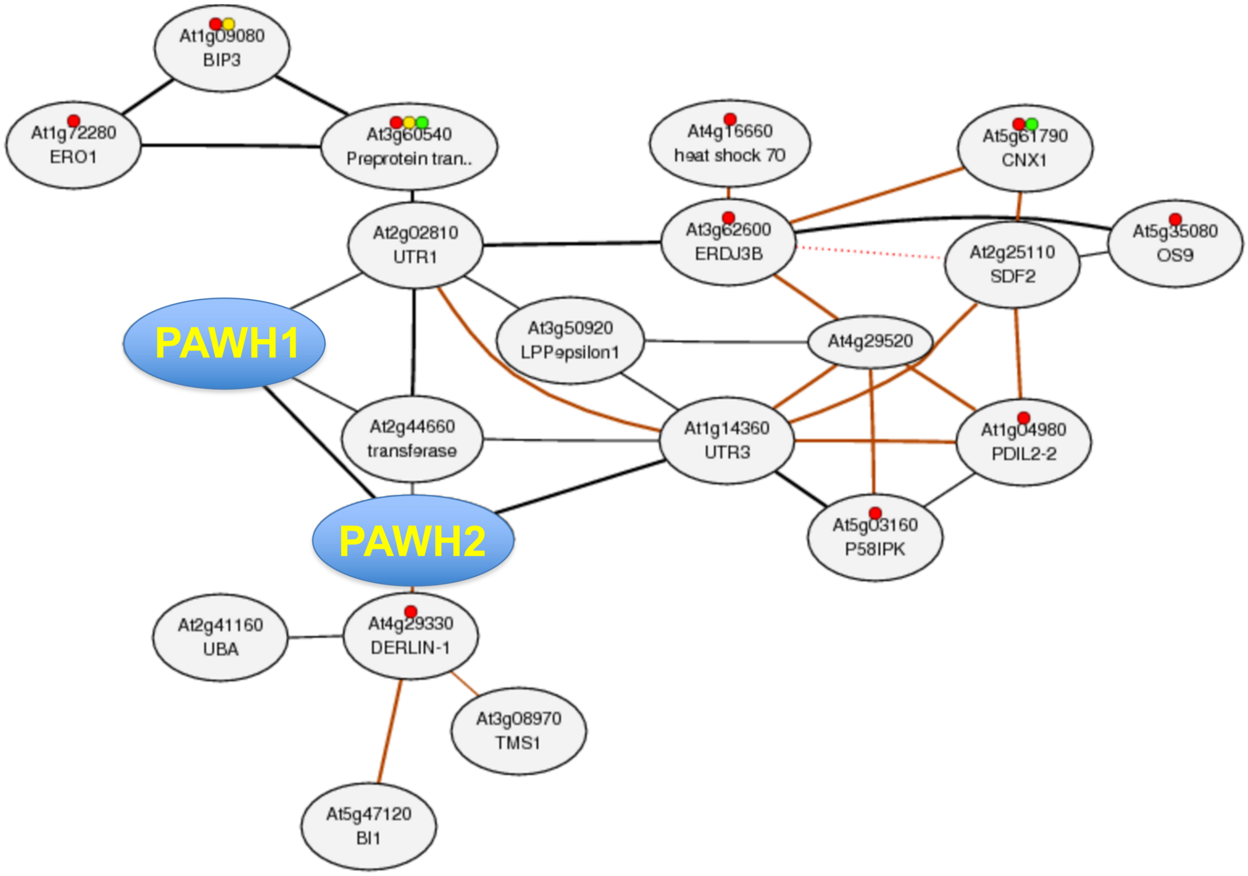

Supplementary Fig. 8. The two *PAWH* genes are coexpressed with known or predicted genes involved in ER-mediated protein folding and protein quality control. Identification of potential *PAWH1/2*-coexpressed genes was performed at ATTEDII (atted.jp[4]) using the gene ID (*AT5G47420*) of *PAWH2* as the query. The image was obtained from the locus page of the search result. Red, yellow, and green dots denote proteins known or predicted to be involved in protein processing in the ER, protein export, and phagosome, respectively. Black thick and thin lines link strongly and weakly coexpressed genes, respectively, orange lines denote coexpressed gene orthologs in other living organisms, while the dotted orange line indicates known protein-protein interactions.

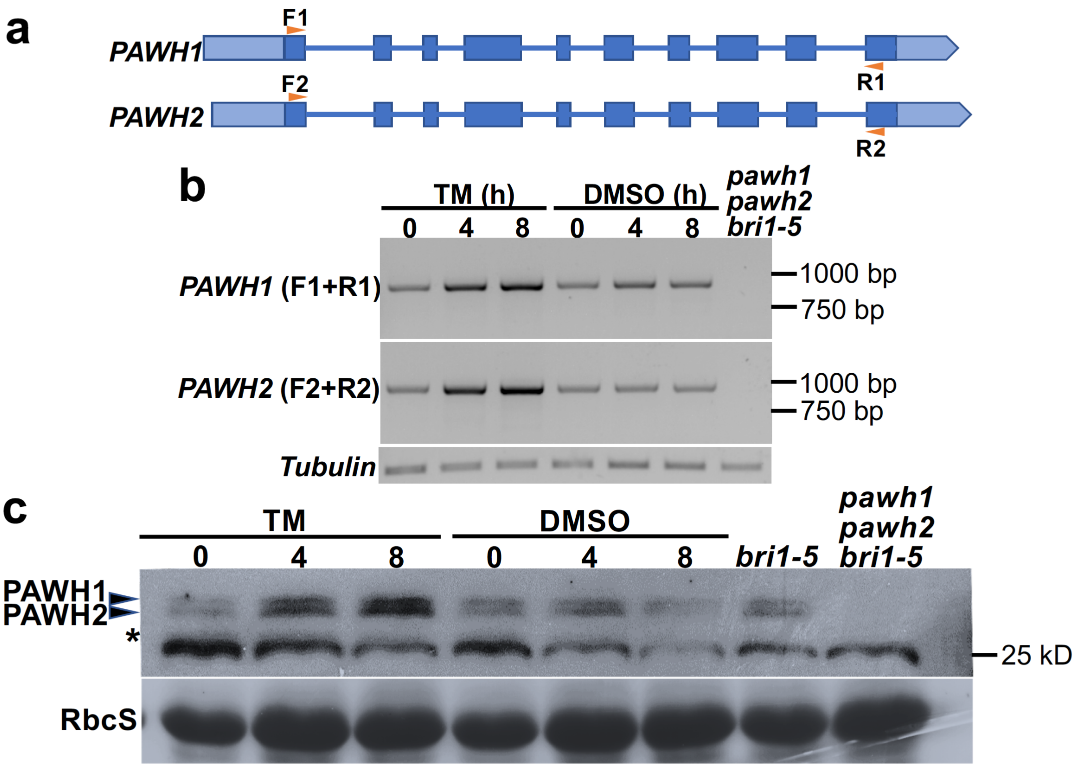

Supplementary Fig. 9. Both *PAWH* genes and their proteins are induced by tunicamycin. **a.** The diagram of the *PAWH1/PAWH2* gene structures. Dark blue bars indicate exons, the light blue bars represent untranslated regions of the first and last exons whereas the thin blue lines denote introns. The orange arrows show the positions of oligonucleotides (see **Supplementary Table 1** for their actual sequences) used for the RT-PCR analysis of the TM-induced gene expression. **b**. Reverse-transcription-PCR analysis of *PAWH1*/*PAWH2* transcript abundance. Total RNAs were extracted from 10-day-old Arabidopsis seedlings treated with or without 5 μg/mL TM or DMSO (as a negative control) and used to generate first strand-cDNAs, which were subsequently used to amplify cDNA fragments of *PAWH1* and *PAWH2* with the indicated primers shown in **a**. The amplified cDNA fragments were separated by agarose gel electrophoresis, stained with ethidium bromide, and photographed into digital images using the Gel Doc™ XR+ gel documentation system (Bio-Rad). The cDNA fragment of *β-Tubulin* was amplified as an internal reference. The positions of DNA size-markers were shown on the right. **c.** Immunoblot analysis of the PAWH protein abundance. The total proteins extracted from 10-day-old Arabidopsis seedlings treated with 5 μg/mL TM or DMSO were separated by SDS-PAGE and analyzed by immunoblotting with a custom-made anti-PAWH antibody or Ponceau Red-staining to show the RbcS bands for the loading control. The asterisk indicates the non-specific cross-reacting band, and the position of the 25 kD size-marker is shown on the right. Source data are provided as a Source Data file.

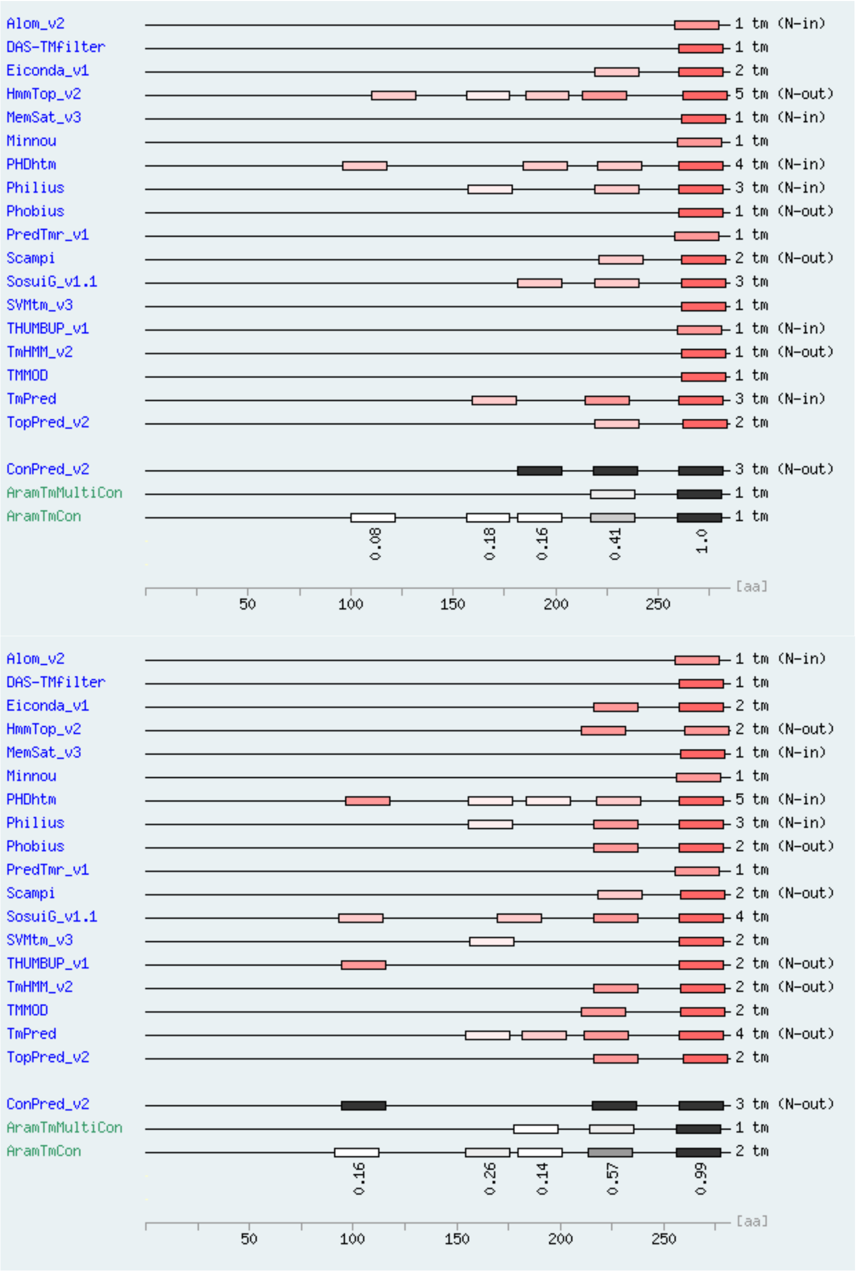

Supplementary Fig. 10. Both PAWH proteins likely have a C-terminal transmembrane domain. The images were obtained from searches at ARAMEMNON (aramemnon.botanik.uni-koeln.de/)[5] using the IDs (At4g17420 and At5g47420) of the two PAWH proteins. A total of 18 bioinformatics programs were used to predict potential transmembrane α-helices, resulting in three consensus predictions with scores. The scores for the predicted C-terminal TM α-helix (LLIQIAIFAFLAYAVILSSLI) and the second most likely TM segment are 1.0 and 0.41 for PAWH1 and 0.99 and 0.57 for PAWH2, respectively.

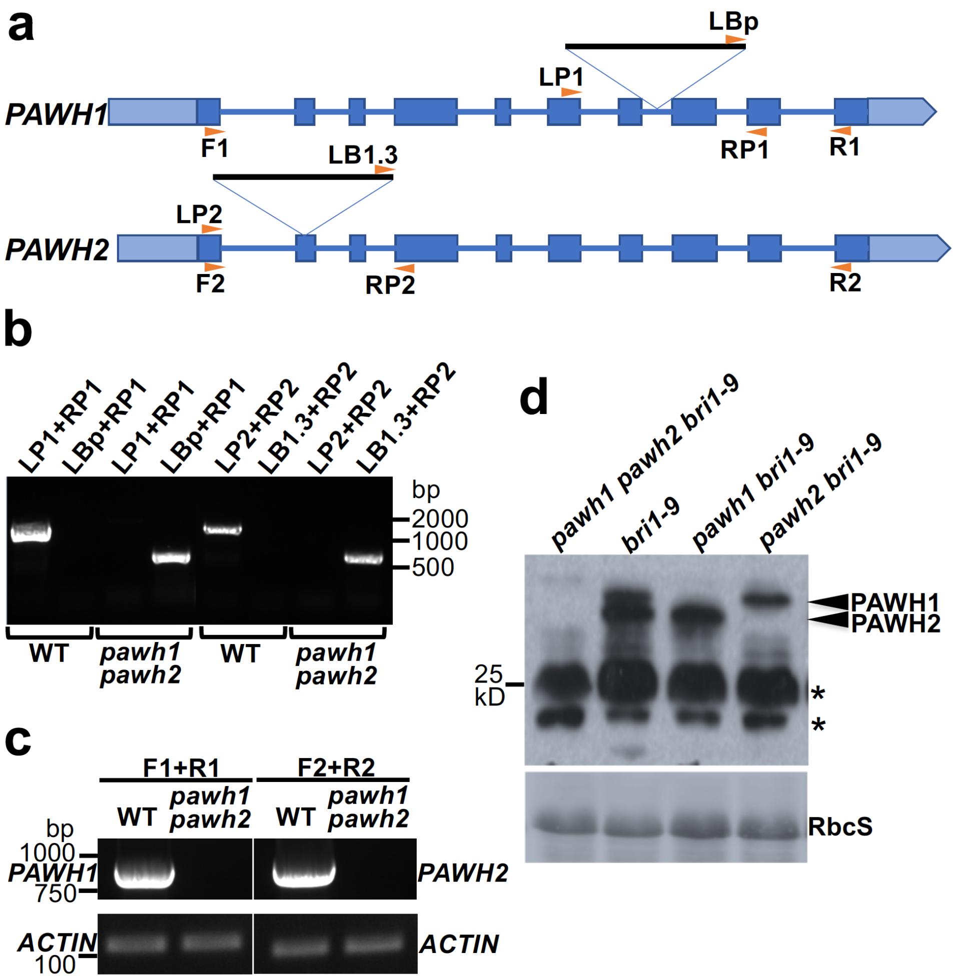

Supplementary Fig. 11. Identification of two T-DNA insertional null mutants of *PAWH1* and *PAWH2* genes. **a.** Diagrams of *PAWH1*/*PAWH2* gene structure showing the locations of the T-DNA insertions. Dark blue bars denote translated exons, light blue bars represent non-translated exons, while the dark blue lines indicate intron sequences. The triangles represent the position of the T-DNA insertions while the orange arrows mark oligonucleotides (see **Supplementary Table 1**) used to genotype the presence and homozygosity of the indicated T-DNA insertions and to analyze the transcript abundance of *PAWH1/2* by RT-PCR. **b.** PCR-based genotyping of the presence and homozygosity of the T-DNA insertions shown in **a**. **c.** RT-PCR analysis of the transcript abundance of *PAWH1*/*PAWH2* (amplified 32 cycles with F1-R1/F2-R2 primers shown in **a**) and *ACTIN* (amplified 28 cycles with primers listed in **Supplementary Table 1**) in the *pawh1 pawh2* double mutant and its wild-type control. **d**. Immunoblot analysis of PAWH1/2 proteins using total proteins extracted from 10-day-old seedlings of the indicated genotypes. The bottom strip shows the Ponceau Red-stained RbcS bands of the same filter for a loading control. The asterisks indicate two non-specific cross-reacting bands. In **b**-**d**, the positions of DNA size markers (bp for base pairs) and protein molecular mass standards are indicated. Source data are provided as a Source Data file.

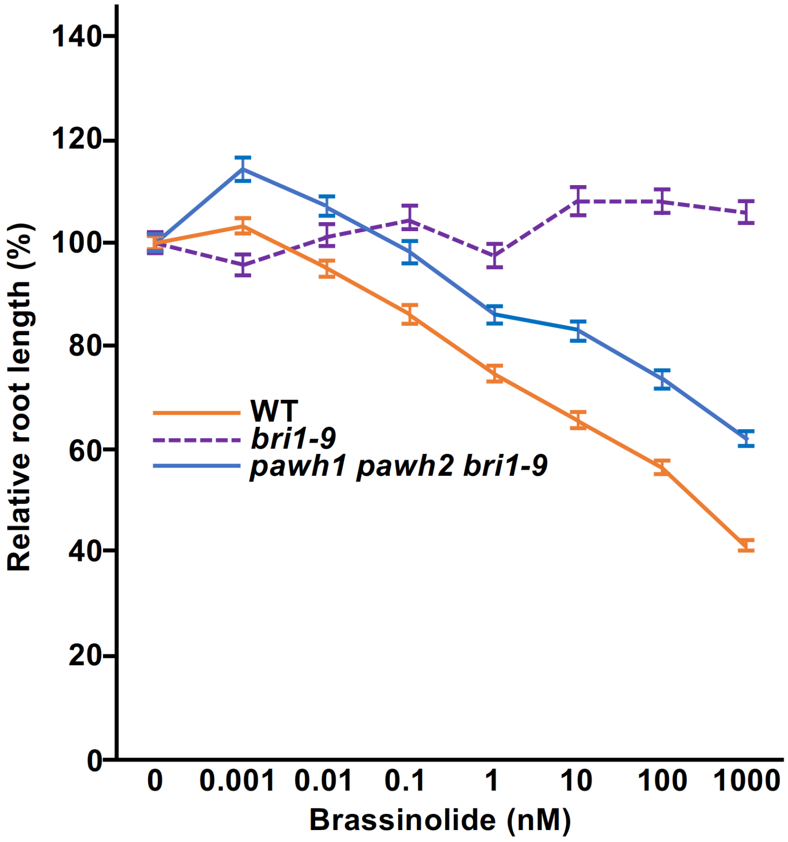

Supplementary Fig. 12. Simultaneous elimination of two PAWH proteins enhances BR signaling of the *bri1-9* mutant. Shown here are the results of the BR-induced root growth inhibition assays. Arabidopsis seedlings germinated and grown on ½ MS medium supplemented with varying concentrations of brassinolide (the most active member of the BR family) were carefully removed from petri dishes after 7-day-growth and photographed into digital images to allow measurement of their root length using ImageJ (http://imagej.nih.gov/ij/). The experiment was performed with three biological replicates each with ~40 seedlings to calculate the average root length for each genotype at each BL concentration. Each data point in the figure indicates the relative value of the average root length of BL-treated seedlings to the average root length of mock-treated seedling of the same genotype. Error bars represent ±SE. Source data are provided as a Source Data file.

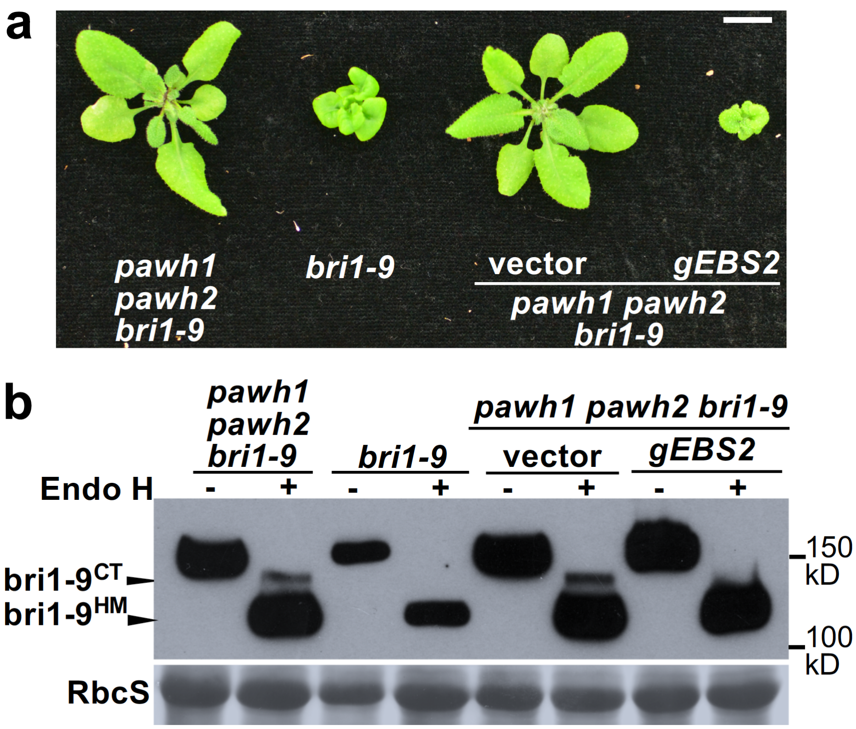

Supplementary Fig. 13. Overexpression of EBS2 nullifies the suppressive effect of the *pawh1 pawh2* double mutation on the *bri1-9* phenotypes. **a.** Photographs of 4-week-old plants grown under a long-day (16h-light/8h-dark) photoperiodic condition. Scale bar = 1 cm. **b.** Immunoblot analysis of the protein abundance and Endo H sensitivity of bri1-9. Total proteins extracted from 4-week-old plants were treated with or without Endo H, separated by 8% SDS-PAGE, and analyzed by immunoblotting with anti-BRI1 antibody. The lower strip is the Ponceau Red-stained immunoblot to control for equal protein loading. The positions of molecular mass standard are indicated by black bars. bri1-9^{CT} and bri1-9^{HM} indicate the bri1-9 forms that are glycosylated with complex-type (CT) and high-mannose-type (HM) glycans, respectively. Source data are provided as a Source Data file.

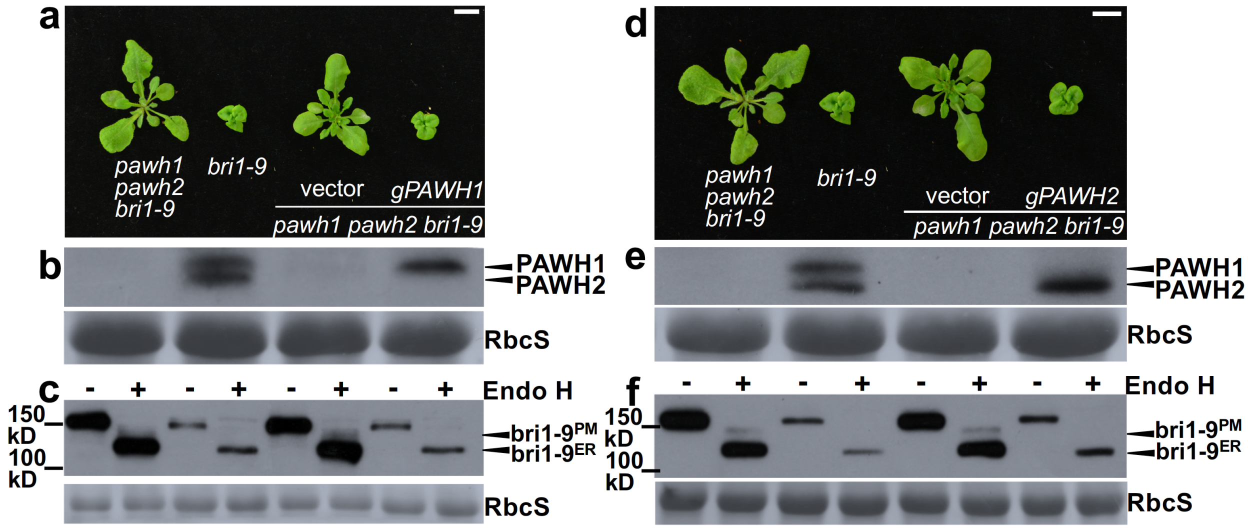

Supplementary Fig. 14. Complementation of the *pawh1 pawh2* double mutation in a *bri1-9* mutant background with a *PAWH1*/*PAWH2* genomic transgene. **a**,**d**. Photographs of 4-week-old soil-grown plants of the indicated genotypes. Scale bar = 1 cm. **b**,**e**. Immunoblot analysis of the PAWH1 (**b**)/PAWH2 (**e**) protein abundance. **c**,**f**. Immunoblot analysis of the protein abundance and Endo-H sensitivity of bri1-9 in total proteins extracted from 2-week-old plants of the indicated genotypes. Total proteins extracted from Arabidopsis plants of the indicated genotype shown in **a** and **d** were treated with (+) or without (-) Endo H, separated by SDS-PAGE, and analyzed by immunoblotting with antibodies against PAWH (**b**,**e**) or BRI1 (**c**,**f**). The lower strips in **c**-**f** show the Ponceau Red-stained RbcS bands to control for equal sample loading. The positions of molecular mass standards are shown on the left. bri1-9^{PM} and bri1-9ER indicate the plasma membrane (PM)- and ER-localized forms of bri1-9, respectively. Source data are provided as a Source Data file.

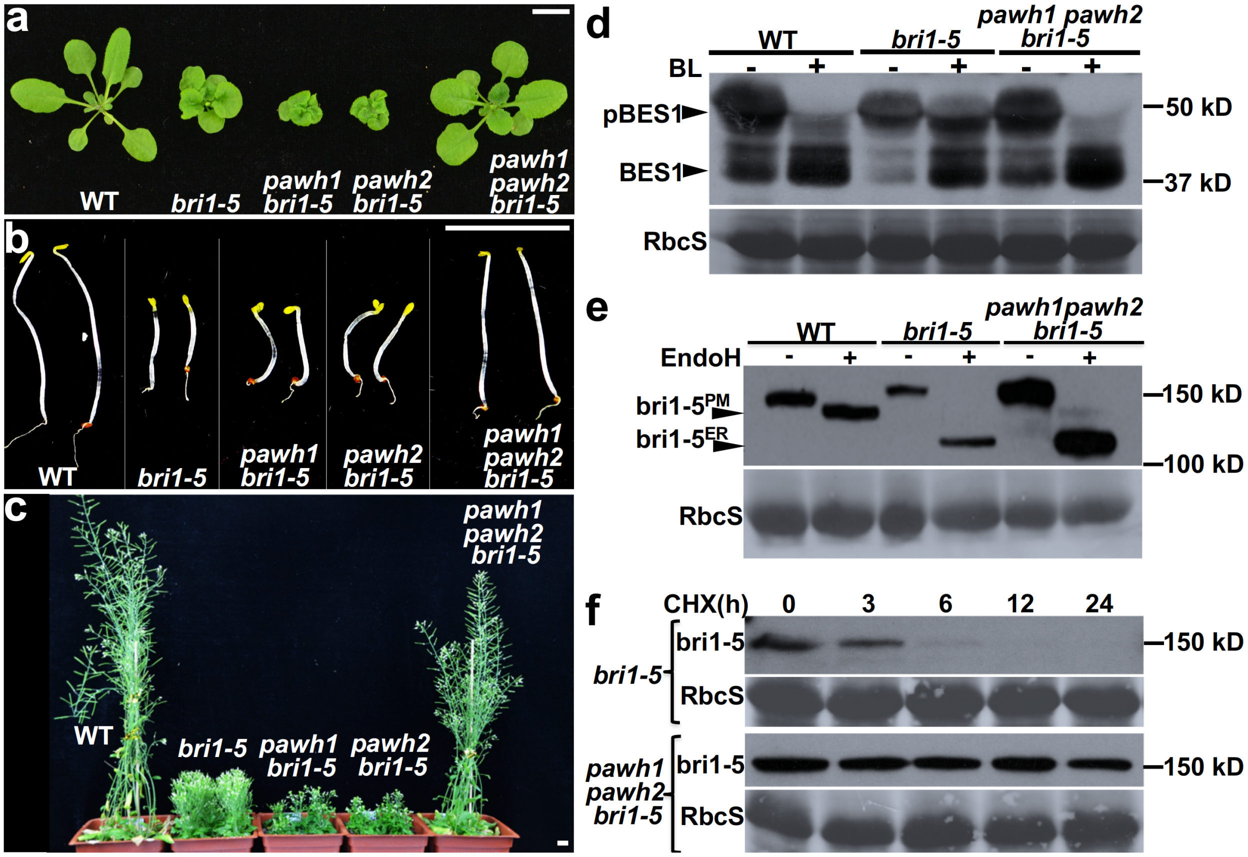

Supplementary Fig. 15. The double T-DNA insertions of the two *PAWH* genes inhibit bri1-5 degradation and suppress the phenotypes of the *bri1-5* mutant. **a**-**c**. Photographs of 3-week-old light-grown seedlings (**a**), 7-day-old dark-grown seedlings (**b**), and 2-month-old soil-grown mature plants (**c**). Scale bar = 1 cm. **d**. Immunoblot analysis of the BL-triggered dephosphorylation of BES1. pBES1 and BES1 indicate the phosphorylated and non-phosphorylated forms of NES1, respectively. **e.** Immunoblot analysis of the protein abundance and Endo-H sensitivity of the wild-type BRI1 and the mutant bri1-5 proteins. bri1-5^{PM} and bri1-5ER denote the PM- and ER-localized forms of bri1-5, respectively. **f.** Immunoblot analysis of the bri1-5 protein stability. Total proteins were extracted from10-day-old seedlings treated with (+) or without (-) 1 μM BL or 180 μM CHX, incubated with (+) or without (-) Endo H, separated by SDS-PAGE, and analyzed by immunoblotting with antibodies to BES1 (**d**) and BRI1 (**e**,**f**). In **d**-**f**, the RbcS-labeled strips are loading controls for the three immunoblotting assays, and the positions of molecular mass standards are shown on the right. Source data are provided as a Source Data file.

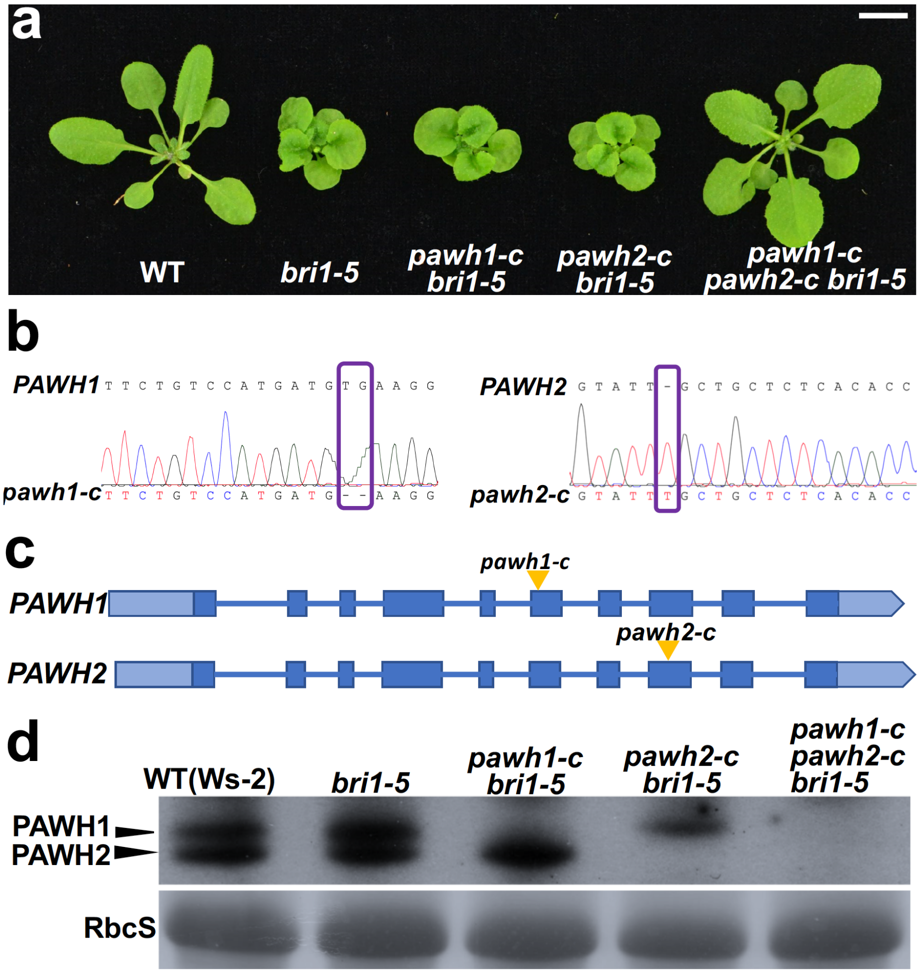

Supplementary Fig. 16. The CRISPR/Cas9-created *pawh1-c pawh2-c* double mutation suppresses the *bri1-5* growth phenotype. **a.** Photographs of 3-week-old light-grown seedlings of the indicated genotypes**. b.** Chromatograms of DNA sequencing showing the two-nucleotide deletion (left) and 1-nucleotide insertion (right) in verified *pawh1-c* and *pawh2-c* mutants, respectively. **c.** Diagrams of the CRISPR/Cas9-created *pawh1-c* and *pawh2-c* mutation sites (indicated by orange arrows) in the 6th and 8th exons of *PAWH1* and *PAWH2* genes, respectively. **d.** Immunoblot analysis of the PAWH1/2 abundance in the indicated genotypes. The lower strip shows the Ponceau Red-stained RbcS bands to control for equal sample loading. Source data are provided in a Source Data file.

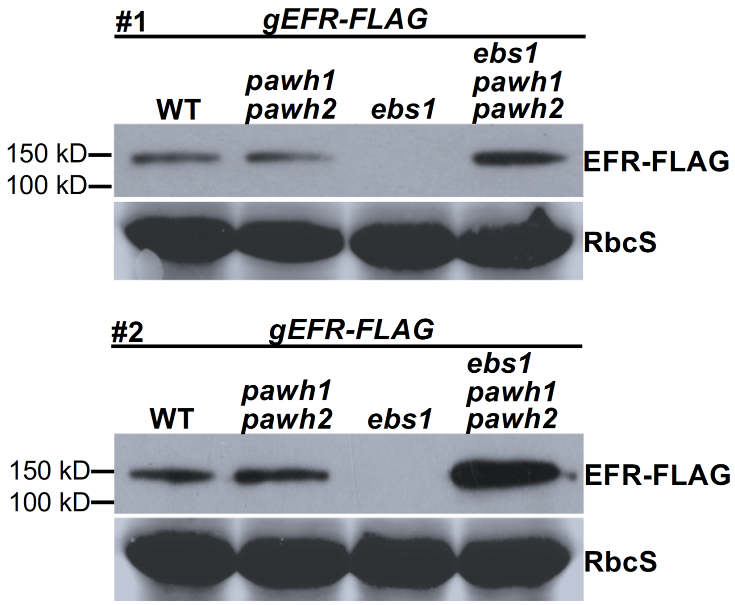

Supplementary Fig. 17. The *pawh1 pawh2* double mutation also stabilizes a misfolded EFR caused by a loss-of-function *ebs1* mutation. Shown here are two independent immunoblot assays of the protein abundance of the transgenically-expressed EFR-FLAG fusion protein. The *gEFR-FLAG* transgene was introduced into wild-type, *ebs1*, *pawh1 pawh2*, and *ebs1 pawh1 pawh2* mutants, and total proteins extracted from several independently-generated 3-week-old transgenic lines were separated by SDS-PAGE and analyzed by immunoblotting with an anti-FLAG antibody. The RbcS strips are used to control equal protein loading. The positions of molecular mass standards are shown on the left. Source data are provided in a Source Data file.

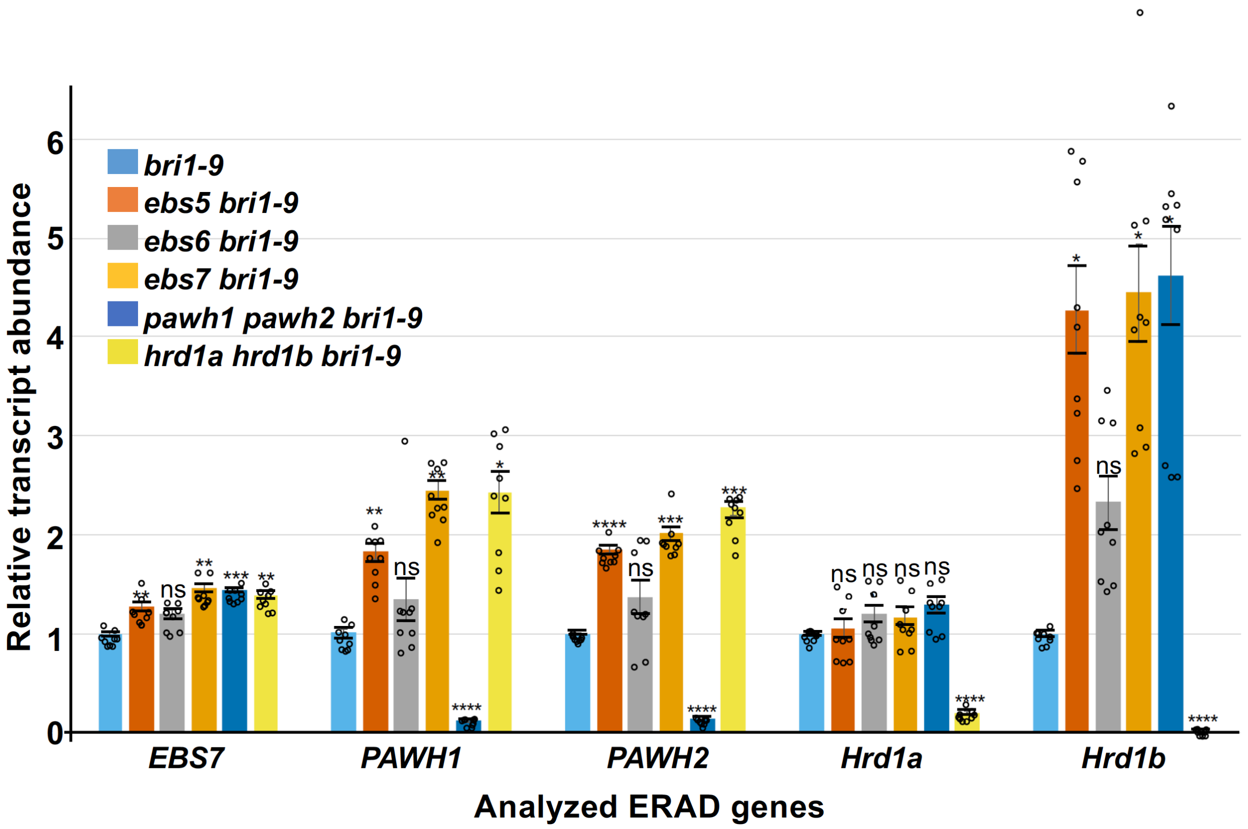

Supplementary Fig. 18. Quantitative real-time PCR analysis of the transcript abundance of *EBS7*, *PAWH1/PAWH2*, and *Hrd1a/1b* genes. Total RNAs isolated from 10-day-old light-grown seedlings were converted into 1st strand cDNAs, which were subsequently used to perform quantitative real-time PCR (qPCR) analysis of the indicated genes with primers listed in **Supplementary Table 1**. Each bar is the relative value of the abundance of a cDNA target in a given genotype to that of the *bri1-9* mutant and is the average result of three biological replicates each with three technical repeats (with individual data points shown as open circles for each colored bar). The *ACTIN8* cDNA was used as an internal reference. Error bars are ±SE, and asterisks indicate significant difference in the transcript abundance of a tested gene between an ERAD mutant and the single *bri1-9* mutant [obtained by student's t-Test performed with the two-tailed t-Test (and nonparametric tests) in GraphPad Prism (https://www.graphpad.com/scientific-software/prism/, ver. 7), ns, non-significant; * $p < 0.05$; ** $p < 0.01$; *** $p < 0.001$; and **** $p < 0.0001$]. Source data are provided as a Source Data file.

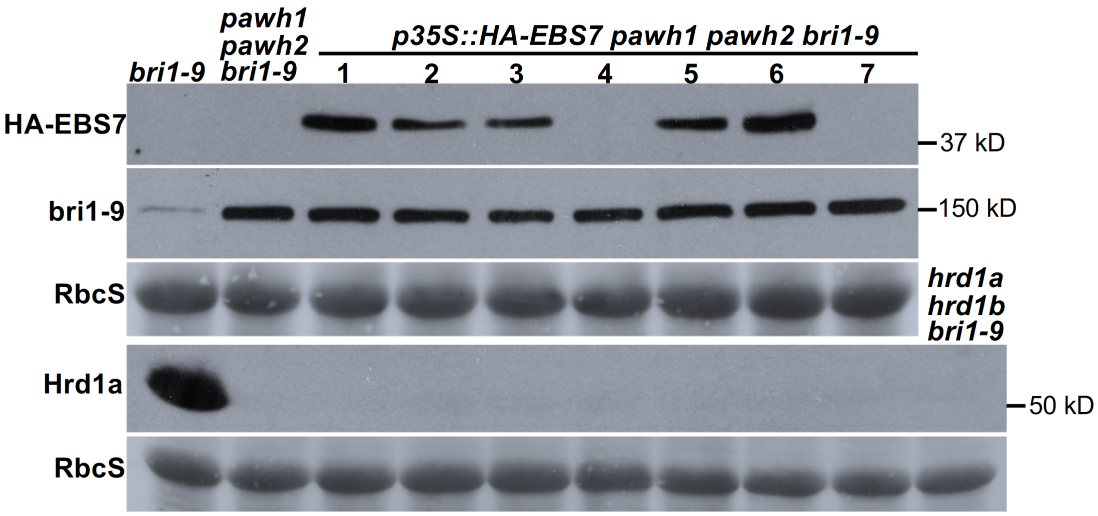

Supplementary Fig. 19. Increased expression of *EBS7* fails to stabilize Hrd1 in *the pawh1 pawh2 bri1-9* mutant. Total proteins extracted from 8-day-old light-grown seedlings of *bri1-9*, *pawh1 pawh2 bri1-9*, and *hrd1a hrd1b bri1-9* mutants, and T2 seedlings of 7 independently-created *p35S::HA-EBS7 pawh1 pawh2 bri1-9* transgenic lines of the same developmental age were separated by SDS-PAGE, and analyzed by immunoblotting with antibodies to HA, BRI1, and Hrd1a. The RbcS-labeled strips were derived from Ponceau Red-stained immunoblots for loading control. The positions of molecular mass standards are shown on the right. Source data are provided as a Source Data file.

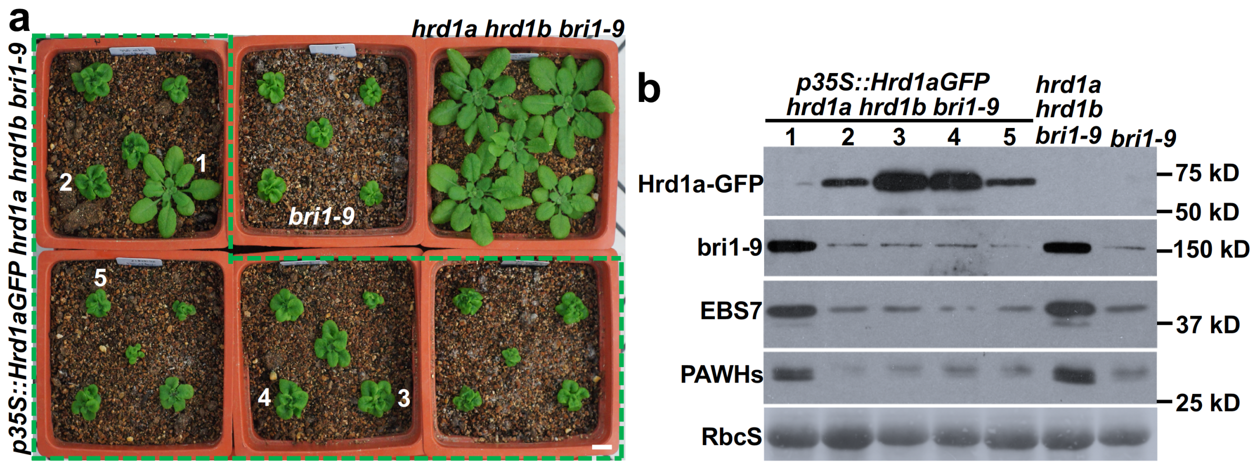

Supplementary Fig. 20. The rescue of growth and biochemical phenotypes of the *hrd1a hrd1b bri1-9* triple mutant by the *p35S::Hrd1-GFP* transgene. **a.** Photographs of 5-week-old soil-grown plants of indicated genotypes. Scale bar = 1 cm. **b.** Immunoblot analysis of total proteins extracted from *bri1-9*, *hrd1a hrd1b bri1-9*, and *p35S::Hrd1a-GFP hrd1a hrd1b bri1-9* transgenic lines with antibodies to GFP, BRI1, EBS7, and PAWH. A strip showing the Ponceau Red-stained RbcS bands was used as a loading control. The positions of molecular mass standards are shown on the right. Source data are provided as a Source Data file.

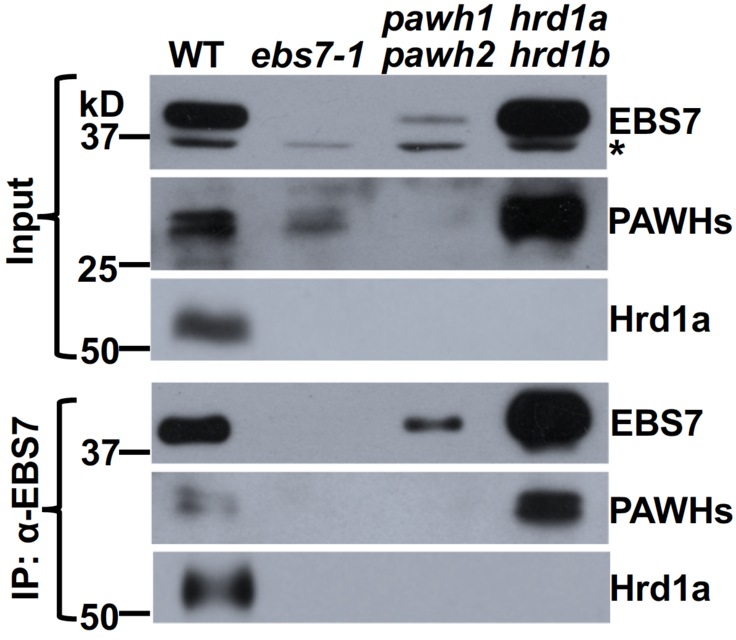

Supplementary Fig. 21. The *hrd1a hrd1b* double mutation has no effect on the EBS7-PAWH interaction. Immunoblot analysis of total proteins and anti-EBS7 immunoprecipitates derived from 10-day-old seedlings of the indicated genotypes with antibodies to EBS7, PAWH, and Hrd1a. The asterisk indicates the non-specific cross-reacting bands, and the positions of molecular mass standards are shown on the left. Source data are provided as a Source Data file.

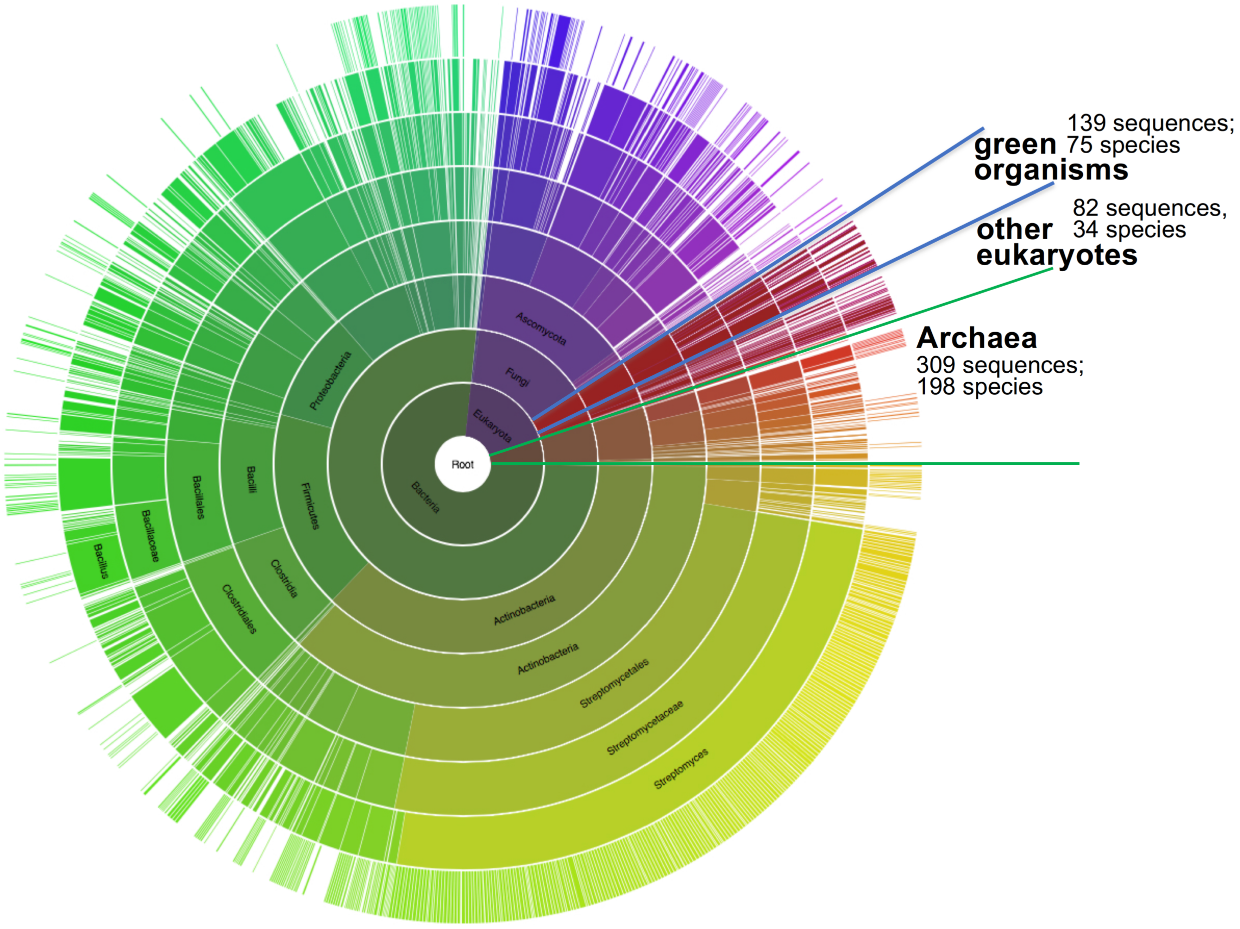

Supplementary Fig 22. The sunburst visualization of the species predicted to have the AIM24 domain-containing proteins in the three domains of the life. The sunburst image was obtained from the Pfam database (pfam.xfam.org)[6] using AIM24 as the enquiry. The numbers of protein sequences and species predicted to contain the AIM24-contaning proteins in green plants (viridiplantae), in the non-fungi/non-viridiplantae other eukaryotes, and in the Archaea domain are shown.

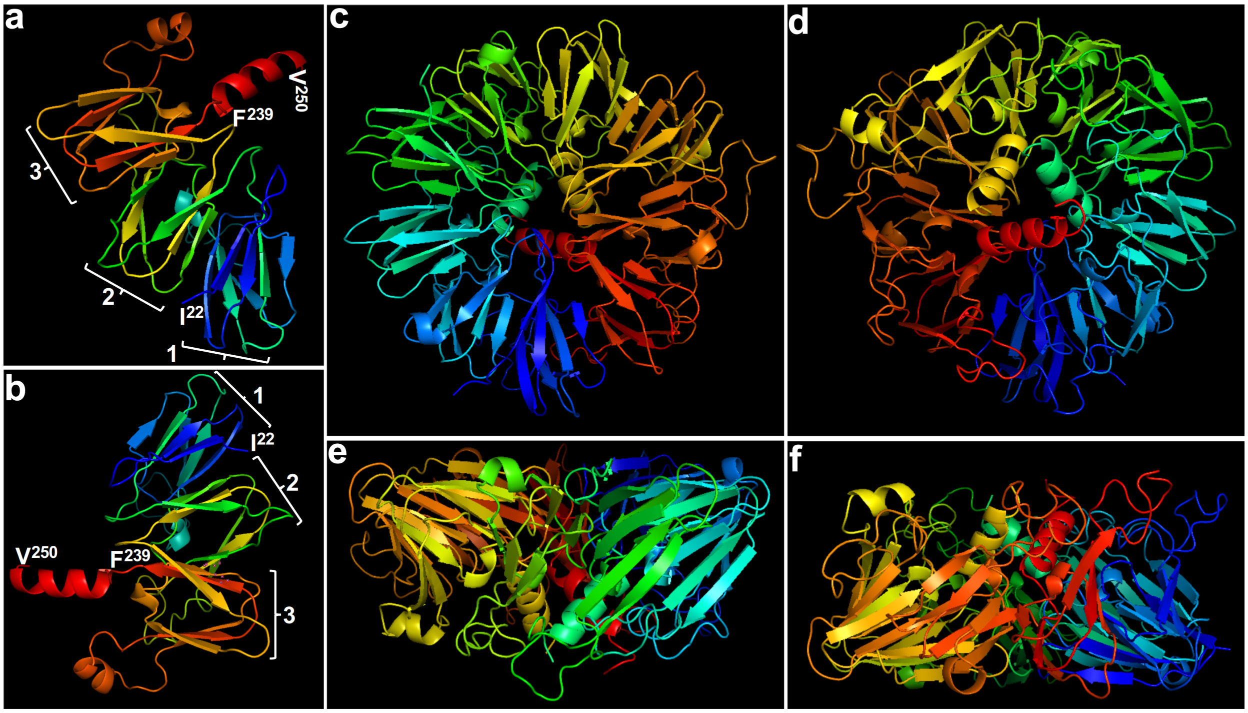

Supplementary Fig. 23. Predicted 3D-structures of PAWH1. The protein sequence (accession number: NP_567527) of PAWH1 was used via an automatic mode at https://www.swissmodel.expasy.org/[7] to search for potential templates, resulting in identification of 18 filtered templates, the tope 12 of which were derived from the structures of two bacterial proteins: 1YOX of the hypothetic protein PA3696 of *Pseudomonas aeruginosa* and 1PG6 of an unknown protein SpyM3_0169 of *Streptococcus pyogenes*. 1PG6 was used to build a 3D model of a monomeric PAWH1 protein (shown in **a**,**b**) while 1YOX was used to build a 3D model of a homotrimeric PAWH1 complex (shown in **c**-**f**). The resulting 3D models were visualized by MacPyMOL (https://pymol.org/2/). **a**,**b**. Two different views of a 3D cartoon model of a monomeric PAWH1 protein with colors indicating positions of amino acids/secondary structures (blue being near the N-terminal end while red indicating proximity to the C-terminus). I^{22} (the 22nd amino acid of PAWH1) is the first residue of the polypeptide used to build the model while F^{239} and V^{250} are the two amino acid residues that define the C-terminal α-helix involved in forming the trimeric PAWH1 complex shown in **c**-**f**. The numeric numbers and square brackets indicate the three structural repeat of a β-sandwich of 3/4 anti-parallel beta-strands. **c**,**d**. The top and bottom views of a 3D cartoon model of a trimeric PAWH1 complex Nine β-sandwich repeats are arranged in a helical pattern to form a closed ring structure (**c**) with the three C-terminal α-helices forming a "three-struct tensegrity" structure (**d**) that might be crucial for the physiological function of PAWH1/2 in plants. **e**,**f**. Two side views of the 3D models shown in **c**,**d**. Both views of the homotrimeric PAWH1 structure model reveal a flat ring-surface that might be involved in interacting with certain protein-binding platforms of EBS7, Hrd1, and/or other yet to be identified plant proteins.

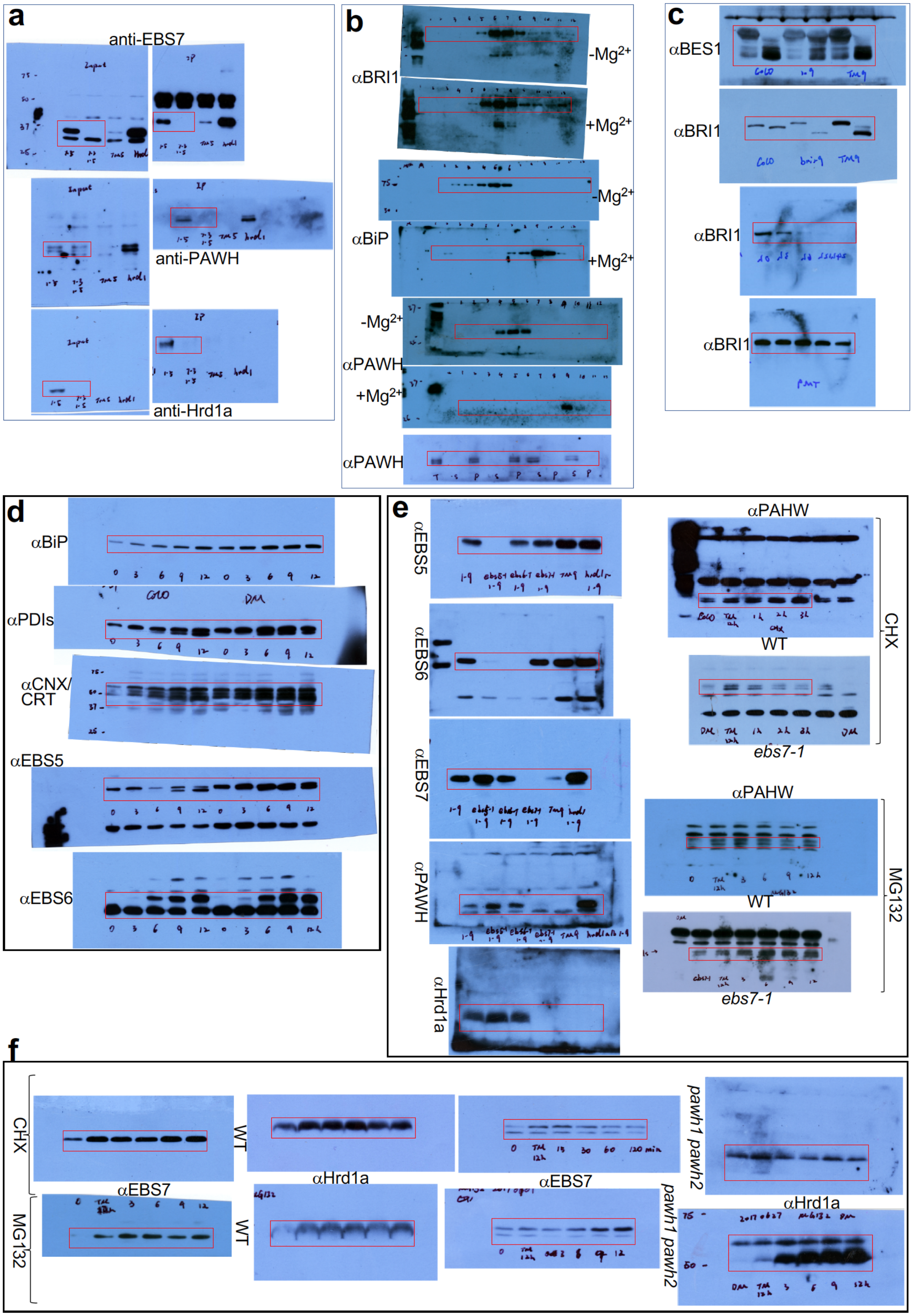
a
anti-EBS7
anti-PAWH
anti-Hrd1a
b
αBRI1
-Mg2+
+Mg2+
αBiP
-Mg2+
+Mg2+
-Mg2+
αPAWH
+Mg2+
αPAWH
c
αBES1
αBRI1
αBRI1
αBRI1
d
αBiP
αPDIs
αCNX/CRT
αEBS5
αEBS6
e
αEBS5
αEBS6
αEBS7
αPAWH
αHrd1a
αPAHW
WT
ebs7-1
CHX
αPAHW
WT
ebs7-1
MG132
f
CHX
MG132
WT
αEBS7
WT
αHrd1a
αEBS7
pawh1 pawh2
αHrd1a
pawh1 pawh2

Supplementary Fig. 24. Full scan images of immunoblots for figures in the main text. **a**. immunoblots for **Fig. 1**. **b**. immunoblots for **Fig. 2b**. **c**. Immunoblots for **Fig. 3**. **d**. Immunoblots for **Fig. 4**. **e**. Immunoblots for **Fig. 5**. **f**. immunoblots for **Fig. 6**. The parts of the scanned full immunoblot images that are presented in the figures are boxed.

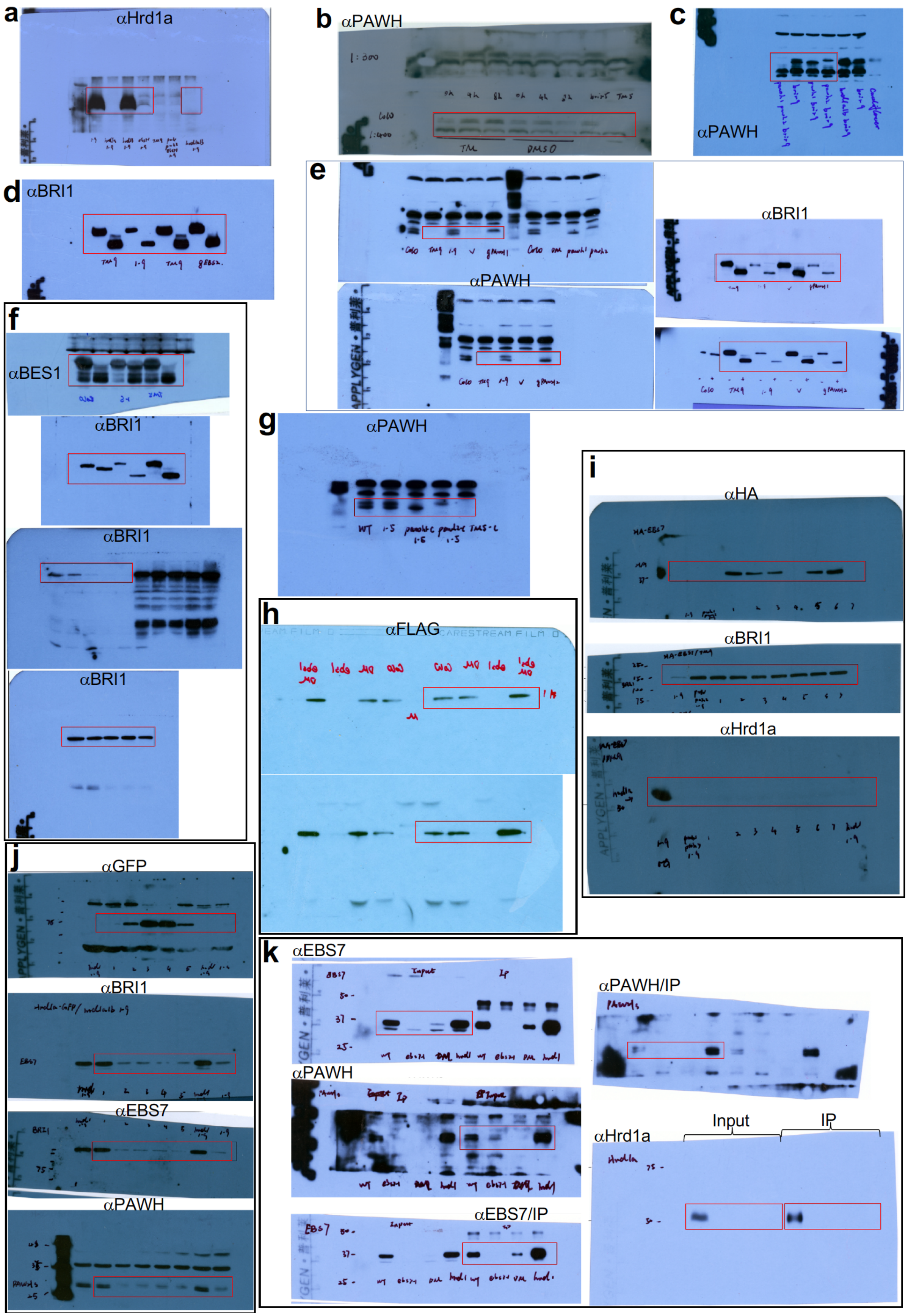
a αHrd1a
b αPAWH
c αPAWH
d αBRI1
e αBRI1
αPAWH
f αBES1
αBRI1
αBRI1
αBRI1
g αPAWH
h αFLAG
i αHA
αBRI1
αHrd1a
j αGFP
αBRI1
αEBS7
αPAWH
k αEBS7
αPAWH/IP
αPAWH
αHrd1a
Input
IP
αEBS7/IP

Supplementary Fig. 25. Full scan images of immunoblots used to generate immunoblots of the supplementary figures. **a**. immunoblots for **Supplementary Fig. 6**. **b**. immunoblots for **Supplementary Fig. 9c**. **c**. immunoblots for generating **Supplementary Fig. 11d**. **d**. Immunoblots for creating **Supplementary Fig. 13b**. **e**. Immunoblots for **Supplementary Fig. 14**. **f**. Immunoblots for creating **Supplementary Fig. 15**. **g**. Immunoblots for **Supplementary 16d**. **h**. Immunoblots for **Supplementary Fig. 17**. **i**. Immunoblots for **Supplementary Fig. 19**. **j**. Immunoblots for **Supplementary Fig. 20b**. **k**. Immunoblots for **Supplementary Fig. 21**. The parts of the scanned full immunoblot images that are used to create the supplementary figures are boxed.

Supplementary Table S1. Oligonucleotides used in this study

Primer Name	Sequence		Digestion Enzyme
	Forward primer	Reverse primer	
**Genotyping**			
PAWH1(CS335767)	GGGATCTCAAAGGCTATTTCG	CGTCAATCTTTAGCTGCTTGG	-
LBp	ATATTGACCATCATACTCATTGC		-
PAWH2(Salk_111654)	TCAACGGAGTTGACAACCTTC	TCTGATCGTTCGAAATTGACC	-
LBb1.3	ATTTTGCCGATTTCGGAAC		-
ebs5-1	TTGTGGTTTAATTGCTTGTTCAC	GCATTACTTTGTGCCACTTCATAAC	SacI
ebs6(Salk_029413)	TCTATGATTTTGGCGTTTTGG	TTCCTTGTTTCTTGTTGCGTC	-
ebs7-1	ATCATAGCTATACGTAACTG	CTTTCCACAACATGCCTTTGATCTG	PstI
ebs7-3	CGGTGCAAGAGCTCCAGTTTGGTAC	ATCTTATGCTGGTATGTC	KpnI
hrd1a(Salk_032914)	CTTGAGCTTATCCGTGACCTG	TGCTACTGTGTTTGCAGATGG	-
hrd1b(Salk_061776)	AGTGGCATCATTTCTGCAAAC	GGAAGGGCTCAGGTGATTAAG	-
ebs1(CS854661)	TTTGGCTCATCAGGAATTGTC	ACGTATGACCTGCAAGGAGAG	-
ebs1-Middle Primer	AACGTCCGCAATGTGTTATTAAGTTGTC		-
**qRT-PCR primers**			
PAWH1	ATGGCTGCTCCGTTTTTCTCTACTC	TCACATTTCAGTAAGGATTAAAGAG	-
PAWH2	ATGGCCGCTCCGTTTTTTTCAACTCC	TCAAACTTCGGTTAAGATCAACGAT	-
EBS7	ATGACGGAAGAACCAGAGAAGGTGT	GGTCTAGTCGTTGGAACATTGTAAG	-
Hrd1a	CATGCATTCAGCAGCAGAGGC	AGCAACAGTCGTTGTCGCCAG	-
Hrd1b	ATGATTCAGCTAAAGGTTTACGCG	CGTGAAGAAGACTGACATTGAAGC	-
β-Tubulin	TTCCAGGTTTGTCACTCGTTG	ATGAAGAAGTGAAGACGGG	-
ACTIN 8	TCAGCACTTTCCAGCAGATG	CTGTGGACAATGCCTGGAC	-
**Yeast two hybrid**			
Y2H-PAWH1	CGC**GAATTC**ATGGCTGCTCCGTTTTTCTCTAC	GAG**GGATCC**TCTTGGATTTTCTCTCATATTTGGG	EcoRI/BamHI
Y2H-PAWH2	CCC**CATATG**ATGGCCGCTCCGTTTTTTTCAAC	CAC**GGATCC**TTTTGGATTCTCTCTCATGTTTGGCG	NdeI/BamHI
**For making transgenic constructs**			
gPAWH1	TATGACCATGATTAC**GAATTC**GCTTCTTAAGACTGACAGGTACAAAATC	GCCAAGCTTGCATGC**CTGCAG**CTAGCAAAGTTAATCATGTGGAAGATG	EcoRI/PstI
gPAWH2	TATGACCATGATTAC**GAATTC**TCGTCAAAAGAGCTTTTGGTTCT	GCCAAGCTTGCATGC**CTGCAG**AAAATTAATCAATAATATCATTAGACATTAATTATT	EcoRI/PstI
GFP-PAWH1	CGC**GGATCC**ATGGCTGCTCCGTTTTTCTCTACTC	CGC**GTCGAC**TCACATTTCAGTAAGGATTAAAGAG	BamHI/SalI
GFP-PAWH2	CGC**GGATCC**ATGGCCGCTCCGTTTTTTTCAACTCC	CGC**GTCGAC**TCAAACTTCGGTTAAGATCAACGAT	BamHI/SalI

EBS7	CG**ACGCGT**AATGACGGAAGAACCAGAGAAG	GG**ACTAGT**ACAGAATAAAATTAAGGAAACG	MluI/SpeI
Hrd1a-GFP	CGG**GGTACC**ATGATTCGACTAAGAAC	GG**ACTAGT**CTCTGCTGCATCAGCAAC	KpnI/SpeI
gEFR-FLAG	CGC**GGTACC**CGTTGATTATTTCTCTTTAAGTGAA	CGC**GGATCC**CATAGTATGCATGTCCGTATTTAAC	KpnI/BamHI
**CRISPR/Cas9 primers**			
PAWH1	GATTGTGTTCTGTCCATGATGTGA	AAACTCACATCATGGACAGAACAC	-
PAWH2	GATTGGGTGTGAGAGCAGCAATAC	AAACGTATTGCTGCTCTCACACCC	-
**For making BIFC construscts**			
NER-PAWH1	CGC**GGATCC**ATGGCTGCTCCGTTTTTCTCTAC	CCG**CTCGAG**CATTTCAGTAAGGATTAAAGAGGACAC	BamHI/XhoI
NER-PAWH2	CGC**GGATCC**ATGGCCGCTCCGTTTTTTTCAAC	CCG**CTCGAG**TCAAACTTCGGTTAAGATCAACGATG	BamHI/XhoI
CER-EBS7	ACGC**GTCGAC**ATGACGGAAGAACCAGAGAAG	CGG**GGTACC**CTACTCTATGTTGTGAAAACC	SalI/KpnI
CER-Hrd1a	ACGC**GTCGAC**ATGATTCGACTAAGAACAT	CGG**GGTACC**CTCTGCTGCATCAGCAACC	SalI/KpnI
**For making PAWH1 and Hrd1a antigen**			
antigen-PAWH1	CGC**GGATCC**ATGGCTGCTCCGTTTTTCTC	CCC**CTCGAG**CCTTTGTCTCAGAACCCCCTCAGC	BamHI/XhoI
antigen-Hrd1a-1	GC**GGATCC**CAAGTGTCAACTGAACCAG	GC**GGTACC**ATCAGCAACCGACTTTCC	BamHI/KpnI
antigen-Hrd1a-2	GC**GGTACC**CAAGTGTCAACTGAACCAG	GC**GTCGAC**ATCAGCAACCGACTTTCC	KpnI/SalI

Supplementary References:

1. Raherison, E.S., Giguere, I., Caron, S., Lamara, M. & MacKay, J.J. Modular organization of the white spruce (Picea glauca) transcriptome reveals functional organization and evolutionary signatures. *New Phytol* **207**, 172-87 (2015).
2. Dereeper, A. *et al.* Phylogeny.fr: robust phylogenetic analysis for the non-specialist. *Nucleic Acids Res* **36**, W465-9 (2008).
3. Winter, D. *et al.* An "electronic fluorescent pictograph" browser for exploring and analyzing large-scale biological data sets. *PLoS One* **2**, e718 (2007).
4. Obayashi, T., Hayashi, S., Saeki, M., Ohta, H. & Kinoshita, K. ATTED-II provides coexpressed gene networks for Arabidopsis. *Nucleic Acids Res* **37**, D987-91 (2009).
5. Schwacke, R. *et al.* ARAMEMNON, a novel database for Arabidopsis integral membrane proteins. *Plant Physiol* **131**, 16-26 (2003).
6. Finn, R.D. *et al.* The Pfam protein families database: towards a more sustainable future. *Nucleic Acids Res* **44**, D279-85 (2016).
7. Waterhouse, A. *et al.* SWISS-MODEL: homology modelling of protein structures and complexes. *Nucleic Acids Res* **46**, W296-W303 (2018).
