# Supplementary material for: PAWH1 and PAWH2 are plant-specific components of an Arabidopsis endoplasmic reticulum-associated degradation complex
Source: Nat Commun. 2019 Aug 2;10:3492. doi: 10.1038/s41467-019-11480-7 (PMC6677890; doi:10.1038/s41467-019-11480-7)
Supplement: Supplementary file 3 — Reporting Summary [file 41467_2019_11480_MOESM3_ESM.pdf]

nature research

Corresponding author(s): Jianming Li

Last updated by author(s): Jul 5, 2019

Reporting Summary

Nature Research wishes to improve the reproducibility of the work that we publish. This form provides structure for consistency and transparency in reporting. For further information on Nature Research policies, see Authors & Referees and the Editorial Policy Checklist .

Statistics

For all statistical analyses, confirm that the following items are present in the figure legend, table legend, main text, or Methods section.

n/a	Confirmed	
☐	☒	The exact sample size (*n*) for each experimental group/condition, given as a discrete number and unit of measurement
☐	☒	A statement on whether measurements were taken from distinct samples or whether the same sample was measured repeatedly
☐	☒	The statistical test(s) used AND whether they are one- or two-sided<br>*Only common tests should be described solely by name; describe more complex techniques in the Methods section.*
☐	☒	A description of all covariates tested
☐	☒	A description of any assumptions or corrections, such as tests of normality and adjustment for multiple comparisons
☐	☒	A full description of the statistical parameters including central tendency (e.g. means) or other basic estimates (e.g. regression coefficient) AND variation (e.g. standard deviation) or associated estimates of uncertainty (e.g. confidence intervals)
☐	☒	For null hypothesis testing, the test statistic (e.g. *F*, *t*, *r*) with confidence intervals, effect sizes, degrees of freedom and *P* value noted<br>*Give P values as exact values whenever suitable.*
☒	☐	For Bayesian analysis, information on the choice of priors and Markov chain Monte Carlo settings
☒	☐	For hierarchical and complex designs, identification of the appropriate level for tests and full reporting of outcomes
☒	☐	Estimates of effect sizes (e.g. Cohen's *d*, Pearson's *r*), indicating how they were calculated

Our web collection on statistics for biologists contains articles on many of the points above.

Software and code

Policy information about availability of computer code

Data collection	The Real-time PCR cycler (CFX96, Bio-Rad) was used to quantitatively measure transcript abundance; Leica SP8 (with LAS AF software, Leica Microsystems) was used for the confocal microscopic examination of the PAWH1/2 localization pattern; The Primer Premier 5 (http://www.premierbiosoft.com/primerdesign/) was used to design oligo-nucleotide primers; The NIH Image J (https://imagej.nih.gov/ij/) was used to measure the root length of Arabidopsis seedlings.
Data analysis	The LC-MS data were processed using the Mascot search engine (v 2.4.0); The target sites for CRISPR/Cas9 were selected using the web program CRISPR-PLANT (http://crispr.hzau.edu.cn/cgi-bin/CRISPR/CRISPR); The Microsoft Excel was used to analyze the stress tolerance of Arabidopsis seedlings and the brassinosteroid sensitivity of different Arabidopsis strains; and the Student's t-Test with the two-tailed t-Test was performed using GraphPad Prism (v 7.0) to determine statistical significance of the qRT-PCR results. The gene expression profiles of the two PAWH genes were obtained from the Arabidopsis eFP browser web site http://bar.utoronto.ca/efp_arabidopsis; The transmembrane domains of the two PAWH proteins were predicted by ARAMEMNON (aramemnon.botanik.unikoeln.de/); The identification of coexpressed genes was performed at ATTEDII at atted.jp; The sunburst image of the wide distribution of the AIM24 domain-containing proteins in the three domains of life was obtained from the Pfam database (pfam.xfam.org); The structure modeling of PAWH1/2 was performed at the SWISS-MODEL (https://www.swissmodel.expasy.org/) using the automatic mode and the resulting models were were visualized by MacPyMOL (https://pymol.org/2/).

For manuscripts utilizing custom algorithms or software that are central to the research but not yet described in published literature, software must be made available to editors/reviewers. We strongly encourage code deposition in a community repository (e.g. GitHub). See the Nature Research guidelines for submitting code & software for further information.

Data

Policy information about availability of data

All manuscripts must include a data availability statement. This statement should provide the following information, where applicable:

- Accession codes, unique identifiers, or web links for publicly available datasets
- A list of figures that have associated raw data
- A description of any restrictions on data availability

The mass spectrometry proteomic data have been deposited to the ProteomeXchange Consortium via the PRIDE partner repository (https://www.ebi.ac.uk/pride/archive/) with the dataset identifier PXD013400. The source data underlying Figs. 1d, 2b,c, 3d,e,f, 4a, 5a,b,c, 6a,b,c,d and Supplementary Figs. 6, 9b,c, 11b,c,d, 12, 13b, 14b,c,e,f, 15d,e,f, 16d, 17, 18, 19, 20b, 21 are provided by a Source Data file. The source data for generating Supplementary Fig. 7 and 8 were obtained from http://bar.utoronto.ca/efp_arabidopsis (using At4g17420 and At5g47420 as queries) and atted.jp (using At5g47420 as query), respectively. All other data are available from the corresponding authors upon a reasonable request.

Field-specific reporting

Please select the one below that is the best fit for your research. If you are not sure, read the appropriate sections before making your selection.

[x] Life sciences [ ] Behavioural & social sciences [ ] Ecological, evolutionary & environmental sciences

For a reference copy of the document with all sections, see nature.com/documents/nr-reporting-summary-flat.pdf

Life sciences study design

All studies must disclose on these points even when the disclosure is negative.

Sample size	Approximately 40 7-day-old seedlings each sample were used for the BL-induced root growth inhibition assay. Sample sizes of other experiences were not pre-specified. No statistical method was used to pre-determine sample sizes.
Data exclusions	No data were excluded from analysis.
Replication	All attempts at replication in each experiment reported in this study were successful.
Randomization	All samples were arranged randomly into experimental groups.
Blinding	Animal experiments were not performed in this study, thus the investigators were not blinded to the experiments reported in this study.

Reporting for specific materials, systems and methods

We require information from authors about some types of materials, experimental systems and methods used in many studies. Here, indicate whether each material, system or method listed is relevant to your study. If you are not sure if a list item applies to your research, read the appropriate section before selecting a response.

Materials & experimental systems

n/a	Involved in the study	
[ ]	[x]	Antibodies
[x]	[ ]	Eukaryotic cell lines
[x]	[ ]	Palaeontology
[x]	[ ]	Animals and other organisms
[x]	[ ]	Human research participants
[x]	[ ]	Clinical data

Methods

n/a	Involved in the study	
[x]	[ ]	ChIP-seq
[x]	[ ]	Flow cytometry
[x]	[ ]	MRI-based neuroimaging

Antibodies

Antibodies used	Anti-EBS5, anti-EBS6, anti-EBS7, anti-BRI1, anti-BES1, anti-CNXs/CRT, anti-Hrd1a, and anti-PAWH are custom-made antibodies. Other antibodies used in this study are commercially available, including anti-BiP (at-95, Santa Cruz Biotechnology), anti-PDI (Rose Biotechnology Inc), anti-GFP (JL-8, 632381, Takara), anti-HA (H9658, Sigma), anti-FLAG (M22000, Abmart), HRP-conjugated goat anti-mouse IgG (1706516, Bio-Rad) and goat anti-rabbit IgG (1706515, Bio-Rad) secondary antibodies.

Validation

The anti-EBS5, anti-EBS6, anti-EBS7 antibodies were previously validated (Su et al., 2011, PNAS 108, 870; Su et al., 2012, Mol Plant 5, 929; Liu et al., 2015, PNAS 112, 12205); the anti-BRI and anti-BES1 antibodies were validated in a previously published study (Mora-Garcia et al., Genes Dev 18, 448-60); the anti-Maize CNXs/CRT was previously validated (Jin et al., 2007, Mol Cell 26:821); the anti-PAWH and anti-Hrd1a antibodies were validated in this study (Supplementary Figure S11 and Supplementary Figure S6, respectively. The commercial antibodies used in this study were certified and validated by manufacturers.
